# Increase and Redistribution of Sex Hormone Receptors in Premenopausal Women Are Associated with Varicose Vein Remodelling

**DOI:** 10.1155/2018/3974026

**Published:** 2018-09-03

**Authors:** Natalio García-Honduvilla, Ángel Asúnsolo, Miguel A. Ortega, Felipe Sainz, Javier Leal, Pedro Lopez-Hervas, Gemma Pascual, Julia Buján

**Affiliations:** ^1^Department of Medicine and Medical Specialities, Faculty of Medicine and Health Sciences, University of Alcalá, Alcalá de Henares. Networking Biomedical Research Center on Bioengineering, Biomaterials and Nanomedicine (CIBER-BBN), Madrid, Spain; ^2^Ramón y Cajal Institute of Sanitary Research (IRYCIS), Madrid, Spain; ^3^University Center of Defense of Madrid (CUD-ACD), Madrid, Spain; ^4^Department of Surgery, Medical and Social Sciences, Faculty of Medicine and Health Sciences, University of Alcala, Alcala de Henares, Madrid, Spain; ^5^Angiology and Vascular Surgery Service, Ruber International Hospital, Madrid, Spain; ^6^Angiology and Vascular Surgery Service, Central University Hospital of Defense-UAH, Madrid, Spain; ^7^General Surgery and Digestive Service, Ramón y Cajal University Hospital, Madrid, Spain

## Abstract

In chronic venous insufficiency of the lower limbs, data show that the clinical manifestation is varicose veins (VVs), and VV epidemiology suggests that sex hormones directly influence disease development through intracellular receptors. This study aimed to determine the presence and localization of oestrogen receptors (ERs), progesterone receptors (PRs), and androgen receptors (ARs) in both healthy and VV wall cells and their relationship with gender. In this study, samples from patients without a history of venous disease (CV) (*n* = 18) and with VV (*n* = 40) were used. The samples were divided by gender: CV women (CVw) = 6, CV men (CVm) = 12, VV women (VVw) = 25, and VV men (VVm) = 15. RT-qPCR and immunohistochemical techniques were performed, and increased ER and PR protein expression was found in VVw in all tunica layers. ARs were localized to the adventitial layer in the CV and were found in the neointima in VVs. mRNA expression was increased for ER and PR in VVw. AR gene expression was significantly decreased in VVm. The increase in the number of these receptors and their redistribution through the wall reinforces the role of sex hormones in varicose vein development.

## 1. Introduction

Varicose veins are the most common form of primary venous insufficiency, with a high prevalence of 20–60% in the Western population [1]. Various theories have attempted to explain why healthy veins become varicose (dilated and tortuous). The primary cause of varicose vein (VV) formation has not been established; however, both valvular dysfunction and venous pressure seem to play key roles in disease onset and progression [2–4]. The force of gravity and the absence of an active venous return mechanism mean that the venous wall components are subjected to intense biomechanical forces that may condition or aggravate functional failure of the venous wall [5, 6]. Macroscopic changes in the veins also occur at a microscopic level through modifications to the extracellular matrix (ECM) and cellular components (especially of smooth muscle cells (SMCs)) [7]. The ECM is the fundamental support network of vascular walls, providing most of their structural properties. Several studies implicate variations in ECM components (collagen fibres, elastic fibres, matrix metalloproteinase (MMP), and glycosaminoglycans) and in SMCs in varicose vein pathology [7–9].

Another key point is the relationship between sex hormones and venous pathology, which primarily focuses on the increased thrombogenic risk they produce [10–12]. Female sex hormones may play a predominant role in varicose physiopathology, especially considering the influence of gender and pregnancy on varicose vein development [13]. How molecular changes occur and how hormones are involved in regulating the venous wall remain to be determined with consideration of the many variables involved, including gender differences in regulating vascular tone, differences in the stimulation or inhibition of cell populations by sex hormones, hormone actions in synthesising ECM products, and actions of hormone receptors and their activation pathways. Previous clinical and epidemiological studies have shown a predominance of varicose veins in women [12, 14, 15]; therefore, this study aimed at verifying whether steroid receptors and progesterone, oestrogen, and androgen receptors are involved in varicose vein development and whether this occurs differently in men and women. Our results show important changes in the localization of these receptors according to histological modifications when vein walls become varicose.

## 2. Patients and Methods

### 2.1. Patients and Samples

Fifty-eight saphenous vein specimens were obtained during surgery from organ donor extraction (controls with no history of venous insufficiency) or varicose vein surgery. Informed consent to participate in this study was obtained from all subjects. The project was approved by the Clinical Research Ethics Committee of the Ruber International Hospital. We divided the study population into two groups according to the sex of patients as follows.

### 2.2. Control Group (CV)

The control group (*n* = 18) was composed of 6 vein specimens harvested from female patients (CVw) [mean age 52.4 ± 4.7, range 43–63 years] and 12 specimens from male patients (CVm) [mean age 61.2 ± 7.8, range 47–69 years].

### 2.3. Varicose Vein Group (VV)

The varicose vein group (*n* = 40) was composed of 25 vein specimens harvested from female patients (VVw) [mean age 43.5 ± 6.6, range 46–62 years] and 15 specimens from male patients (VVm) [mean age 59.6 ± 7.3, range 53–71 years]. Segments of the saphenous vein were obtained at the time of extraction from patients with primary venous insufficiency. The Classification System for Chronic Venous Disorders (CEAP) based on clinical, aetiologic, anatomic, and pathophysiologic data was applied previously to the venous extraction [16].

Immediately after procurement, the specimens were placed in sterile minimum essential medium (MEM) and stored at 4°C and then transferred to the laboratory where they were divided into two fragments, one for microscopy (immunohistochemistry) and the other for storage at −80°C in RNAlater solution until RNA extraction and RT-PCR analysis.

### 2.4. Immunohistochemical Analysis

For immunohistochemical analyses, vein specimens were fixed in 4% paraformaldehyde solution, embedded in paraffin, and cut into 5 *μ*m slices using a microtome (Microm, Barcelona, Spain). The sections were then deparaffinized, hydrated, and equilibrated in PBS (pH 7.4). We used anti-oestrogen, anti-progesterone, and anti-androgen receptor antibodies with specific secondary antibodies (Table 1). The antigen-antibody reaction was detected by peroxidase-labelled avidin-biotin procedures and avidin-alkaline phosphatase (Sigma-Aldrich, St. Louis, MO, USA). The chromogenic substrate contained diaminobenzidine (DAB) for progesterone receptors (PRs) and oestrogen receptor (ER) alpha and alkaline chromogenic substrate for androgen receptors (AR). All protocols followed those of Ortega et al. [13]. After immunostaining, the tissue sections were examined under a light microscope (Zeiss, Jena, Germany). Positive cells were blind-counted under a light microscope (Zeiss) in 4 areas of 0.5 mm^2^ per patient (40 high-power fields per group) by two authors. All values were expressed as the means ± SEM.

### 2.5. qRT-PCR

Tissue fragments of 1 cm^2^ were obtained from the control and varicose veins, immersed in RNAlater solution (Ambion, Austin, TX, USA), and stored at −80°C until use. RNA was extracted through guanidine-phenol-chloroform isothiocyanate procedures using TRIzol (Invitrogen, Carlsbad, CA, USA). RNA was recovered from the aqueous phase and precipitated by adding isopropanol and incubating overnight at −20°C. RNA integrity was checked using a 1% (*w*/*v*) agarose gel and quantified by spectrophotometry. Complementary DNA was synthesised by reverse transcription using 200 ng of the total RNA with oligo (dT) primers (Amersham, Fairfield, USA) and the enzyme Moloney murine leukaemia virus reverse transcriptase (M-MLV RT, Invitrogen). cDNA was amplified by PCR using the following primers: ER sense 5′ GTG GGC GTT CCA AAT GAA AGC CAA G 3′ and antisense 5′ GAG CGC CAG ACG AGA CCA ATC ATC A 3′ at 60°C; PR sense 5′ CCC CAC GGC CAG CAG GTG CCC TAC T 3′ and antisense 5′ GAG CGC CAG ACG AGA CCA ATC ATC A 3′ at 55°C; and androgen receptor (AR) sense 5′ TAC CAG CTC ACC AAG CTC CT 3′ and antisense 5′ GTC TCA CTG GGT GTG GAA AT 3′ at 60°C. The constitutive gene glyceraldehyde 3-phosphate-dehydrogenase (GAPDH) (primers: sense 5′ TCA CCA TCT TCC AGG GA 3′ and antisense 5′ CAC AAT GCC GAA GTG GT 3′ at 60°C) was used as a control.

The RT-qPCR mixture contained 5 *μ*l of inverse transcription product (cDNA) diluted at 1 : 20, 10 *μ*l of iQ SYBR Green Supermix (Bio-Rad Laboratories, Hercules, CA, USA), and 1 *μ*l (6 *μ*M) of each primer, for a final reaction volume of 20 *μ*l. RT-qPCR was performed in a Fast 7500 Applied Biosystems instrument. Samples were subjected to an initial stage of 10 min at 95°C. The cDNA amplification conditions were 40 cycles of 95°C for 15 s, 60°C (the same annealing temperature for each first pair) for 30 s, and 72°C for 1 min. Fluorescence was determined at the end of each cycle. Gene expression was normalized against the expression recorded for the reference gene GAPDH.

### 2.6. Statistical Analysis

GraphPad Prism® 6.0 was used to perform ANOVA, Bonferroni's test (one-tail), and Student's *t*-test for unpaired data. Data are expressed as the mean ± SEM. The following *p* values were considered statistically significant: ^∗^*p* < 0.05, ^∗∗^*p* < 0.01, and ^∗∗∗^*p* < 0.001.

## 3. Results

### 3.1. Protein Analysis

#### 3.1.1. Oestrogen Receptors (ERs)

**Figure 1:**
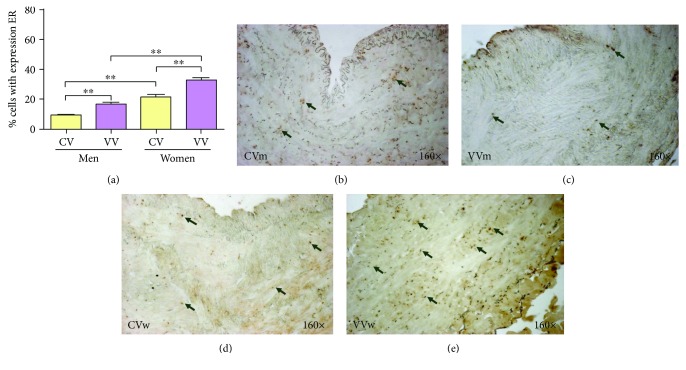
(a). Percentage of cells positively expressing the oestrogen receptor (ER) alpha among the study groups (CV = control vein, VV = varicose vein). (b-e). ER immunodetection images for the four analysis groups. The brown colour indicates the precipitate that correlates with ER protein expression. ^∗∗^*p* < 0.01.

All vein samples stained positive for oestrogen receptor alpha in the vein wall. Oestrogen receptors (alpha) were localized in the cell nuclei in the three vascular wall layers (intima, media, and adventitia) in the healthy and varicose vein groups (Figure 1).

Quantifying the ER-positive cells showed that the mean was 9.03 ± 0.55 in the CVm group and 16.23 ± 1.38 in the VVm group, which was a statistically significant difference (^∗∗^*p* = 0.005). In the women, the mean number of positive cells was 21.13 ± 2.06 in the CVw group and 32.66 ± 1.84 in the VVw group (^∗∗^*p* = 0.0047). Among the control groups, the CVw group was significantly increased compared with the CVm group (^∗∗^*p* = 0.005) and the pathological group (^∗∗^*p* = 0.0012) (Figure 1(a)).

The results obtained show a clear increase in alpha oestrogen receptors in the venous walls of women with varicose veins. This increase in expression was homogenous in the three tunica layers of the varicose vein, distributed intensely throughout the wall.

#### 3.1.2. Progesterone Receptors (PRs)

**Figure 2:**
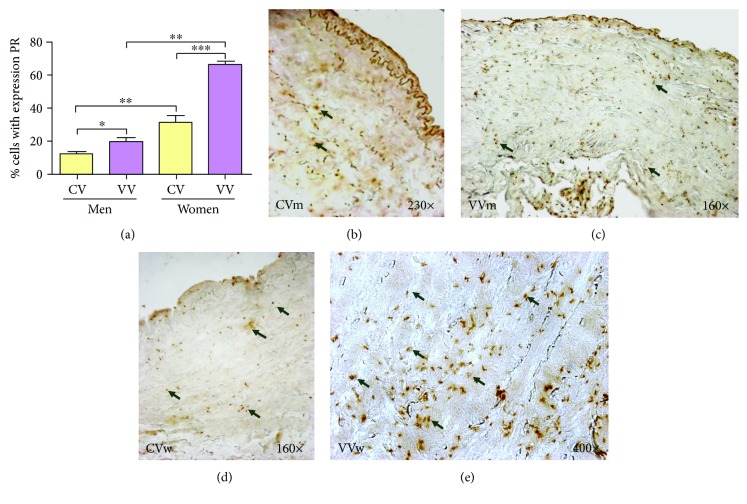
(a). Percentage of cells positively expressing the progesterone receptor (PR) in the study groups (CV = control vein, VV = varicose vein). (b–e). PR immunodetection images from the four analysis groups. The brown colour indicates the precipitate that correlates with PR protein expression. ^∗^*p* < 0.05, ^∗∗^*p* < 0.01, and ^∗∗∗^*p* < 0.001.

In the pathological varicose veins, PR expression showed the same trend as ER expression, and this expression was more abundant than that in healthy veins, especially in women (Figure 2).

Quantitative analyses showed that the PR-positive cell mean was 11.40 ± 1.96 (mean ± SEM) in the CVm group and significantly increased to 19.16 ± 2.83 in the VVm group (^∗^*p* = 0.0221). In the CVw group, the positive cell mean was 30.71 ± 4.67, which significantly differed from the VVw group mean of 66.16 ± 2.01 (^∗∗∗^*p* = 0.0006). Significant ratios were also observed between the CV groups (^∗∗^*p* = 0.0042) and the VV groups (^∗∗^*p* = 0.0021). Overall, the pathological veins showed greater expression of the PR marker, and within groups, women showed the most PR-positive cells (Figure 2(a)).

In women with varicose veins, the presence of progesterone receptors is two times higher than that in the group without varicose veins. The expression of progesterone receptors was observed in all tunica layers of the venous wall.

#### 3.1.3. Androgen Receptors (ARs)

**Figure 3:**
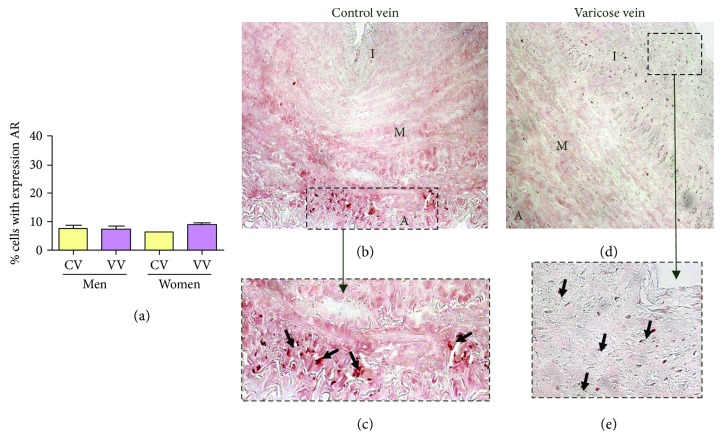
(a). Percentage of cells positively expressing the androgen receptor (AR) in the study groups (CV = control vein, VV = varicose vein). (b–e) AR immunodetection images in the four analysis groups. The red colour indicates the precipitate that correlates with AR protein expression.

Androgen receptors were confined to the nuclei of a few cells in the adventitial layers of healthy veins (Figures 3(b) and 3(c)); however, in varicose veins, the positive cells were mainly located in the neointimal layer (Figures 3(d) and 3(e)).

The percentage of AR-positive cells was low in all groups (CVm = 7.48 ± 1.08, VVm = 7.24 ± 1.13, CVw = 6.02 ± 0.24, and VVw = 8.56 ± 0.79). No significant differences were found in the values among the different populations (Figure 3(a)).

### 3.2. Genetic Analysis

#### 3.2.1. Oestrogen Receptor (ERs)

**Figure 4:**
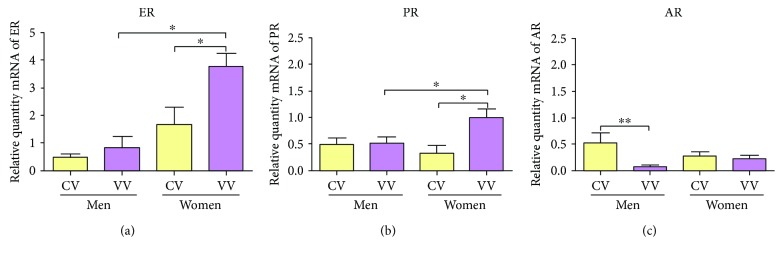
mRNA levels for the oestrogen receptor (ER) alpha (a), progesterone receptor (PR) (b), and androgen receptor (AR) genes (c) quantified by RT-qPCR. The results were normalized to that of the reference gene GAPDH and are provided in arbitrary units. CV = control vein and VV = varicose vein. ^∗^*p* < 0.05 and ^∗∗^*p* < 0.01.

The mean expression of ERs was 0.51 ± 0.11 RQ (relative quantity mRNA) in the CVm group, 0.88 ± 0.37 RQ in the VVm group, 1.67 ± 0.63 RQ in the CVw group, and 3.63 ± 0.46 RQ in the VVw group. Significant differences were established between the CVw and the VVw groups (^∗^*p* = 0.047) as well as between the pathology groups (^∗^*p* = 0.016) (Figure 4(a)).

#### 3.2.2. Progesterone Receptors (PRs)

Gene expression of the PRs showed a mean ± SEM of 0.51 ± 0.11 RQ in the CVm group and 0.53 ± 0.10 RQ in the VVm group, with no significant differences between the groups. For the women, the average was 0.33 ± 0.14 RQ in the CVw group and 1.01 ± 0.16 RQ in the VVw group, indicating a statistically significant increase (^∗^*p* = 0.049). A statistically significant increase was also found between the VVm and the VVw groups, ^∗^*p* = 0.044 (Figure 4(b)).

#### 3.2.3. Androgen Receptors (ARs)

AR expression was detected in all patients studied.  Figure 4(c) reveals that only in men was the AR mRNA expression significantly higher in the control veins than in the varicose veins (^∗∗^*p* = 0.004), while no significant differences were observed in AR expression between the CVw and the VVw groups. The mean AR expression was 0.53 ± 0.19 RQ in the CVm group, 0.02 ± 0.01 RQ in the VVm group, 0.28 ± 0.07 RQ in the CVw group, and 0.23 ± 0.67 RQ in the VVw group (Figure 4(c)).

## 4. Discussion

In this study, we observed an increase in sex hormone receptors under varicose vein conditions in both genders. Furthermore, we want to highlight the importance of the redistribution of these receptors through the venous wall. This redistribution could be related to the pressure change in varicose veins and the histological remodelling of the vein wall.

Population age is one of the main factors affecting the presence of chronic disease. The venous system is especially affected by changes in ageing, and the effects are unequal between the genders [8]. Per the Framingham study, the annual occurrence of varicose veins is 2.6% in women and 1.9% in men [17].

This finding remains controversial because some authors consider that truncal varicose veins are equally common in men and women, while others indicate that importance lies in the reticular varicose veins and telangiectasias that are more common in women [17–19]. In women, key events are directly related to the appearance of varicose veins, such as pregnancy and menopause, which are influenced by age and hormones [19, 20]. During menopause, sex hormones play important roles, as they are involved in numerous pathologies [21]. Oestrogen receptors are present in the endothelium, SMCs, and some adventitial cells in the femoral veins [22]. Our results showed that in the varicose veins of women around menopause, there was a two-fold increase in the number of ER^+^ cells and ER gene expression compared to those in the veins of healthy women. The number of ERs was about two times lower in men. These results agree with the studies of Mashiah et al. [23], who used a different methodology for the evaluation of oestrogen receptors. Several pathological anatomy studies have indicated that the human saphenous vein stains positive for progesterone receptors in both genders [24]. The staining is uniformly distributed in the SMCs of the media and subendothelium, adventitial fibroblasts, and vasa vasorum [25, 26]. Additionally, RT-qPCR has demonstrated the presence of progesterone receptor mRNA [26]. Our study showed the presence of PR in all three tunica layers (endothelium, media, and adventitia) at levels twice as high in varicose patients compared to that in healthy patients of both genders. Even in the vein walls of varicose veins of women, the increases in the number of PR-positive cells and PR mRNA expression were three-fold compared to those in healthy veins of women. Using the same technique, the presence of ER-delta4 isoform mRNA and protein has also been demonstrated in both mammary arterial cell and human saphenous vein cell cultures, and this expression was not located in the nucleus, which may be related to effects from nongenomic oestrogens [27]. The same author previously demonstrated ER mRNA presence in human saphenous vein SMCs, and this receptor can be activated [28].

Other authors have shown oestrogen and progesterone receptors in the human internal saphenous vein [24, 25, 28, 29]. Bergqvist et al. [25] described the positive expression of PR and ER in all samples from fertile women, but ER expression was not observed in samples from postmenopausal women or from men. Mashiah et al. [23] and Krasiński et al. [29] found positive ER expression in the ECM and SMC nuclei and PR in the nuclei of the SMC and subendothelial cells. Mashiah et al. [23] found that both receptors were expressed in the adventitial vasa vasorum cells. These authors described a nonuniform distribution of these cells, with more oestrogen and progesterone receptors (for both genders) in the varicose samples than in the controls. In the varicose samples, the numbers of oestrogen and progesterone receptors were higher in the varicose segments than in the nonvaricose segments in both men and women. The number of oestrogen and progesterone receptors in varicose veins was significantly higher in women when studied with semiquantitative methods. Our results coincide in part with those of other authors [23, 25–28] in terms of the increase in homologous receptors in varicose pathology. However, we found the presence of nuclear receptors at the adventitial level. The findings of previous authors are limited to the presence of receptors in the endothelium of the vasa vasorum. In addition, our results show protein expression and correlation with gene expression.

The direct effects of oestrogen on the human saphenous vein wall have been described in a few studies. Greater methylation of the ER*α* gene was found in samples from older individuals, implying lower activity [30, 31]. The authors hypothesized that the methylation associated with ER*α* gene inactivation could play a role in vascular tissue ageing. 17*β*-Oestradiol affects venous contractility, increasing the vacuum caused by ET-1 but not vasodilating it [32]. 17*β*-Oestradiol inhibits the Ca^2+^-dependent vasoconstriction of saphenous veins in vitro. It also inhibits the vasoconstriction mediated by increased K*^+^* as well as the contraction induced by CaCl*_2_*, suggesting that E2 possibly interferes with Ca^2+^ channels [33]. These authors also described that oestradiol independently inhibits Ca^2+^. Oestradiol dose-dependently inhibits vasoconstriction induced by phorbol dibutyrate (PDB) in media without Ca^2+^ and in SMCs depleted of Ca^2+^, where PGF_2_*α* produces contraction and oestradiol relaxes SMCs. This study suggests that part of the relaxing effect of E2 is independent of the Ca^2+^ channel blockade. Under supraphysiological conditions, 17*β*-oestradiol dilates the umbilical artery and vein [34]. 17*β*-Oestradiol does not appear to decrease *α*-adrenergic system activity in SMCs in vivo [35]. 17*β*-Oestradiol markedly dilates the femoral vein in healthy sows under physiological conditions and in ovariectomized sows, both dependently and independently of the endothelium [22].

Other studies described a lower venous distensibility in contraction under low hormone levels (oophorectomized rats), with the response at rest equal to that of the control veins. In this model, hormone replacement partially restored the loss of compliance [36, 37]. Thus, high oestrogen levels in menopausal women have been associated with more varicose veins and greater venous distensibility [38, 39]. Endodiode-dependent vasodilation (dorsal vein of the hand) was improved in postmenopausal women with hormone replacement therapy compared with controls who received no substitution treatment [40], suggesting the relevance of these hormones.

Conversely, the study of androgen receptors in regard to its possible relationship with venous pathology has not been a research interest. Kendler et al. [41] described dysregulation of androgenic receptors in the varicose veins of men compared with healthy ulnar veins. These authors tried to relate the increase in the levels of sex hormones in the blood of varicose patients with the dysregulation of hormone receptors. Our results coincide with these findings, showing downregulated AR gene expression in men with varicose veins. Our immunohistochemical study shows, for the first time, the different locations of these receptors. In control saphenous veins (men and women), androgen receptors were found at the level of the adventitial tunica. However, in varicose veins, the location of these receptors was changed, occurring at the level of the neointima.

Some studies show that inhibition of androgen receptors can occur in the presence of high levels of progesterone [42]. Our results show changes in the location of the hormonal receptors and a greater activity of ER and PR at the adventitial level and of AR in the neointima of patients with varicose veins. This change in expression could be related to a compensatory effect via an increase in the hydrostatic and tangential pressure of the varicose veins. The increase in receptors could stimulate the proliferation of vascular cells responsible for morphological changes such as the increased diameter, thickness, and tortuosity characteristics of varicose veins, as seen by some authors in in vitro studies of endothelial cells and smooth muscle cells [43].

## 5. Conclusions

The above results show the effects of sex hormones on the vascular system, especially in women. Varicose veins in women are associated with increased oestrogen and progesterone receptors in all tunica layers of the vein wall. However, the location of androgenic receptors was observed in the control patients only at the level of the adventitial tunica. In varicose veins, this expression showed redistribution to the neointima. The overexpression of oestrogen and progesterone receptors and androgenic receptor redistribution in the varicose vein wall reinforce the hypothesis that hormones are involved in varicose vein pathophysiology. These studies demonstrate the necessity of developing new strategies for premenopausal women directed at preventing the development of varicose veins.

## Figures and Tables

**Table 1:** Primary and secondary antibodies used and their dilutions.

Antigen	Species	Dilution	Provider
Oestrogen receptor (ER) alpha	Rabbit	1 : 100	RM-9101 (Neomarkers, Fremont, CA, USA)
Progesterone receptor (PR)	Rabbit	1 : 100	RM-9102 (Neomarkers, Fremont, CA, USA)
Androgen receptor (AR)	Mouse	1 : 25	Ab9474 (Abcam, Cambridge, UK)
Anti-rabbit IgG	Mouse	1 : 1000	RG-96 (Sigma-Aldrich, St. Louis, MO, USA)
Anti-mouse IgG	Goat	1 : 300	Polyclonal (Sigma-Aldrich, St. Louis, Missouri, USA)

## Data Availability

The data used to support the finding of this study are available from the corresponding author upon request.
